# Preoperative Risk Assessment: A Poor Predictor of Outcome in Critically ill Elderly with Sepsis After Abdominal Surgery

**DOI:** 10.1007/s00268-020-05742-5

**Published:** 2020-08-30

**Authors:** Anne C. M. Cuijpers, Marielle M. E. Coolsen, Ronny M. Schnabel, Susanne van Santen, Steven W. M. Olde Damink, Marcel C. G. van de Poll

**Affiliations:** 1 Department of Surgery, Maastricht University Medical Centre+, Postbus 5800, 6202 AZ Maastricht, The Netherlands; 2 Intensive Care Department, Maastricht University Medical Centre+, Postbus 5800, 6202 AZ Maastricht, The Netherlands; 3 Faculty of Health Medicine and Life Sciences, School for Nutrition and Translational Research in Metabolism (NUTRIM), Maastricht University, Universiteitssingel 40, 6229 ER Maastricht, The Netherlands

## Abstract

**Background:**

Postoperative outcome prediction in elderly is based on preoperative physical status but its predictive value is uncertain. The goal was to evaluate the value of risk assessment performed perioperatively in predicting outcome in case of admission to an intensive care unit (ICU).

**Methods:**

A total of 108 postsurgical patients were retrospectively selected from a prospectively recorded database of 144 elderly septic patients (>70 years) admitted to the ICU department after elective or emergency abdominal surgery between 2012 and 2017. Perioperative risk assessment scores including Portsmouth Physiological and Operative Severity Score for the enumeration of Mortality (P-POSSUM) and American Society of Anaesthesiologists Physical Status classification (ASA) were determined. Acute Physiology and Chronic Health Evaluation IV (APACHE IV) was obtained at ICU admission.

**Results:**

In-hospital mortality was 48.9% in elderly requiring ICU admission after elective surgery (*n* = 45), compared to 49.2% after emergency surgery (*n* = 63). APACHE IV significantly predicted in-hospital mortality after complicated elective surgery [area under the curve 0.935 (*p* < 0.001)] where outpatient ASA physical status and P-POSSUM did not. In contrast, P-POSSUM and APACHE IV significantly predicted in-hospital mortality when based on current physical state in elderly requiring emergency surgery (AUC 0.769 (*p* = 0.002) and 0.736 (*p* = 0.006), respectively).

**Conclusions:**

Perioperative risk assessment reflecting premorbid physical status of elderly loses its value when complications occur requiring unplanned ICU admission. Risks in elderly should be re-assessed based on current clinical condition prior to ICU admission, because outcome prediction is more reliable then.

## Introduction

The global population is aging. Worldwide, the number of people aged over 60 is expected to double to 1.8 billion and the group of people aged 80 years or over are expected to increase threefold to 425 million by 2050. This process of aging is most advanced in Europe [1]. With the general population aging, the number of elderly demanding for elective abdominal surgery is increasing [2]. Consequently, the number of elderly patients requiring an intensive care unit (ICU) admission because of complications following major abdominal surgery increases [3]. The term ‘elderly’ has not been universally defined, but mortality after major abdominal surgery in people over the age of 70 is substantially higher than in younger patients [4]. Reliable perioperative risk assessment in elderly may improve patient selection and clinical decision making [5, 6].

When elderly patients are considered for an unplanned ICU admission due to complications after elective surgery, perioperative risk assessment based on the premorbid physical status is often used as a reference for outcome prediction, without taking into account the impact of an invasive surgical procedure and a subsequent severe complication as a second hit on the resulting physical and functional reserve capacity. In clinical practice however, elderly who seemed well-functioning and fit for surgery based on perioperative risk assessment and clinical impression at the outpatient clinic often do not recover from critically illness.

Several perioperative risk assessment tools are available of which American Society of Anaesthesiologists physical status classification (ASA), Portsmouth Physiological and Operative Severity Score for the enumeration of Mortality (P-POSSUM) and Acute Physiology and Chronic Health Evaluation (APACHE) are most frequently used [7]. Measures of geriatric frailty are increasingly recognized as a predictor for adverse health related outcomes in the elderly and might be valuable in perioperative assessment [8–10].

Although frequently referenced, general perioperative risk assessment including premorbid physical status often appears insufficient when elderly become critically ill after surgery and are in need of an ICU admission [5, 6, 11].

The aim of this study was to evaluate if initial perioperative risk assessment is a reliable predictor of mortality in critically ill elderly requiring ICU admission due to severe complications after elective surgery.

## Material and methods

### Study design

The study was performed at the ICU department of the Maastricht University Medical Centre (MUMC+), a tertiary referral centre in the Netherlands. Ethical approval was obtained by the local Medical Ethical Committee of the MUMC+ (METC 2017-0279). Informed consent was waived, because of the retrospective nature of the study using anonymized data obtained from routine care.

Patients were retrospectively selected from a prospectively recorded database of all patients admitted to the ICU with sepsis. Admission with sepsis was defined as any admission to the ICU clinically coded as infection with at least one organ dysfunction [12]. Eligible for inclusion were all patients aged over 70 with sepsis after elective abdominal surgery requiring an unplanned ICU admission or with sepsis requiring emergency abdominal surgery and subsequent ICU admission between 2012 and 2017. Patients requiring prolonged postoperative monitoring and supportive care are admitted to a Post-Anaesthesia-Care-Unit (PACU) for a maximum of 24 h. These patients were only included when transferred to the ICU, which was also defined as an unplanned ICU admission.

### Data collection

Demographic and clinical data as well as outcome data were retrieved from the electronic patient files. Data on age, gender, reason for admission, co-morbidities, ICU mortality and in-hospital mortality were recorded.

### Perioperative risk, comorbidity scores and frailty assessment

ASA physical status was obtained directly from the preoperative anaesthesiology screening records [13]. P-POSSUM mortality score was calculated based on data gathered from the electronic patient files [14, 15]. APACHE IV scores at the time of ICU admission were retrieved from the Dutch National Intensive Care Evaluation (NICE) [16]. Revised cardiac risk index (RCRI) [17], modified frailty index (mFI) [18], and Charlson Comorbidity Index (CCI) [19] were used to quantify premorbid comorbidities and frailty.

In patients with multiple surgical interventions, the clinical status during the preoperative screening of the first operation was recorded. Hence for patients with an elective index operation, risk indices were calculated based on outpatient data, which enabled us to explore the relation between premorbid status and outcomes of complicated surgery. In patients with an emergency index operation, data were used that were obtained as close as possible prior to surgery. ASA physical status, P-POSSUM mortality scores and APACHE IV are affected by rapid changes in physiology and acute illness, whereas the other scores primarily assess chronic co-morbidities and functional state. For this reason, the association between ASA physical status, P-POSSUM mortality scores and APACHE IV was assessed for elective and emergent patients separately, whereas the association between all other scores and outcome was assessed in the entire cohort.

### Outcome variables

The primary outcome was in-hospital mortality. Secondary outcome measure was ICU mortality.

### Statistical analysis

Q–Q plots and Kolmogorov–Smirnov tests were used to check for normality. *χ*^2^ and Fisher exact test were used for categorical values. Independent samples *t* tests and Mann–Whitney *U* tests were used for continuous variables, according to normality. Two-tailed *p* < 0.05 were considered statistically significant. Normally distributed valuables are displayed as mean (± standard deviation). When not normally distributed, values are displayed as median [interquartile range]. Predictive accuracy of ASA physical status, P-POSSUM mortality score and APACHE IV was evaluated using the area under the curve (AUC) of the receiver operating characteristic (ROC) curve as a measure of discrimination and Hosmer–Lemeshow statistics as a measure of calibration. Statistical analysis was performed using SPSS 23 (IBM Corp. Armonk, NY).

## Results

### Baseline characteristics

**Fig. 1:**
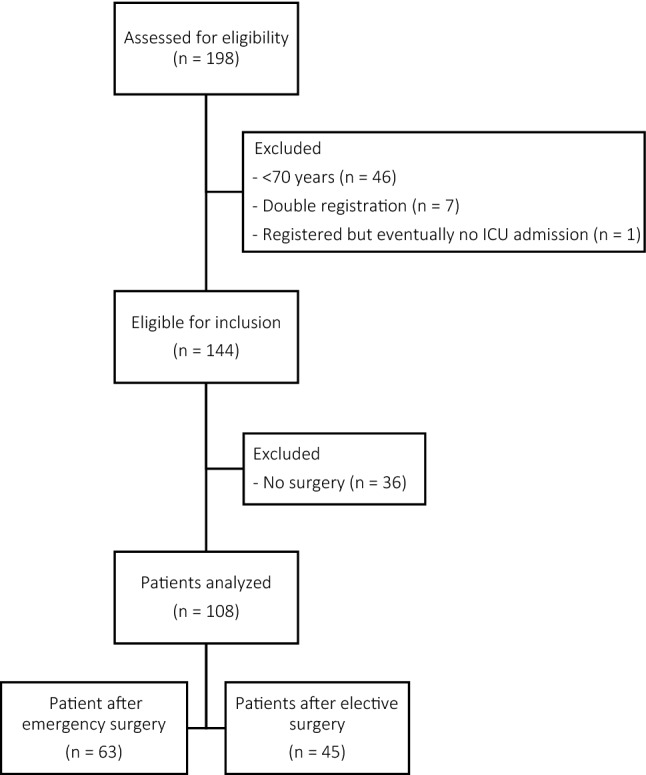
Flowchart of patient selection. Software: Microsoft Word

A total of 108 patients meeting the inclusion criteria between January 2012 and December 2017 were identified. The inclusion flowchart is presented in Fig. 1. In total 45 patients were admitted after elective abdominal surgery and 63 after emergency abdominal surgery. Mean age was, respectively, 76 and 78 with a male to female ratio of 2 to 1 in both groups. Main reason for ICU admission was abdominal sepsis after intestinal perforation, bowel obstruction or anastomotic leakage. Baseline characteristics are presented in Table 1.

**Table 1:** Baseline characteristics

	Total population *N* = 108	Elective surgery *N* = 45	Emergency surgery *N* = 63
Gender			
Male	70 (64.8%)	30 (66.7%)	40 (63.5%)
Age (year)	77.2 (± 5.0)	76.0 (± 4.6)	78.0 (± 5.1)
Moment of ICU admission*			
Preoperative	9 (8.4%)	0 (0%)	9 (14.5%)
Immediately postoperative	46 (43%)	7 (15.6%)	39 (62.9%)
Postoperative from ward	52 (48.6%)	38 (84.5%)	14 (22.5%)
With repeated surgery	19 (17.8%)	16 (35.6%)	3 (4.8%)
Without repeated surgery	33 (30.8)	22 (48.9%)	11 (17.7%)
Cause of sepsis			
Anastomotic leakage	15 (13.9%)	11 (24.4%)	4 (6.3%)
Biliary complications	10 (9.3%)	8 (17.8%)	2 (3.2%)
Intestinal perforation^1^	32 (29.6%)	5 (11.1%)	27 (42.9%)
Bowel obstruction^2^	22 (20.4%)	1 (2.2%)	21 (33.3%)
Pancreatitis	1 (0.9%)	0 (0%)	1 (1.6%)
Intra-abdominal abces^3^	2 (1.9%)	2 (4.4%)	0 (0%)
Bowel ischemia	10 (9.3%)	3 (6.7%)	7 (11.1%)
Wound infection	1 (0.9%)	1 (2.2%)	0 (0%)
Fistula	1 (0.9%)	0 (0%)	0 (0%)
Intra-abdominal bleeding	1 (0.9%)	3 (6.7%)	0 (0%)
Postoperative sepsis of other cause^4^	11 (10.2%)	10 (22.2%)	1 (1.6%)
Repeated surgery during hospitalization**	62 (57.9%)	31 (68.9%)	31 (50%)
Mortality rates			
ICU mortality	40 (37%)	18 (40%)	22 (34.9%)
In-hospital mortality	53 (49.1%)	22 (48.9%)	31 (49.2%)
Postoperative survival (days)^∑^	23 [6–24]	31 [10–146]	14 [3–42]
ASA physical status^†^			
I	0 (0%)	0 (0%)	0 (0%)
II	45 (43.7%)	21 (47.7%)	24 (40.7%)
III	45 (43.7%)	23 (52.3%)	22 (37.3%)
IV	13 (12.6%)	0 (0%)	13 (22.0%)
P-POSSUM mortality score^‡^	13.24 [4.52–40.98]	4.97 [1.85–12.32]	21.81 [9.50–53.99]
APACHE IV^§^	92.5 (± 28.95)	98.69 (± 32.04)	88.69 (± 26.49)

Data displayed as absolute number (%), mean (SD) and median [IQR]

*ICU* Intensive care unit, *ASA physical status* American Society of Anaesthesiologists physical status classification, *P-POSSUM* Portsmouth Physiological and Operative Severity Score for the enumeration of Mortality, *APACHE IV* Acute Physiology and Chronic Health Evaluation IV

^*^N = 107 (elective 45, emergency 62), **N = 102 (elective 44, emergency 62), ^∑^ in patients not surviving hospitalization, ^†^*N* = 103 (elective 44, emergency 59), ^‡^*N* = 97 (elective 37, emergency 60), ^§^*N* = 84 (elective 32, emergency 52)

^1^Both iatrogenic and spontaneous perforation based on infection or malignancy

^2^Obstruction based on adhesions, malignancy of volvulus

^3^Abscess postoperative or in combination with malignancy

^4^Pneumosepsis or urosepsis

### Perioperative risk assessment and mortality

**Fig. 2:**
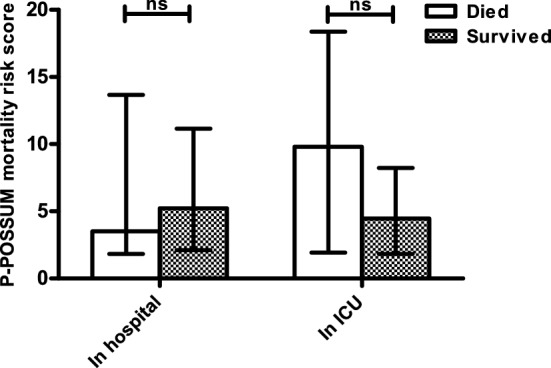
P-POSSUM mortality prediction scores in elderly patients admitted to the ICU for a septic complication after elective surgery. Software: GraphPad Prism 5

ICU mortality was 40% in critically ill elderly requiring ICU admission after complications following elective surgery, increasing to an in-hospital mortality of 48.9% with a median postoperative survival of 31 days. When P-POSSUM mortality scores were compared in patients with complications after elective surgery who survived to hospital discharge and patients who did not, no significant difference in predicted mortality was seen (Mann Whitney *U*, *p* = 0.951) (Table 2A, Fig. 2). Furthermore, no statistically significant differences in P-POSSUM mortality score were observed between patients who did and did not survive the ICU admission (Mann Whitney *U*, *p* = 0.445) (Table 2B, Fig. 2). Also regarding the ASA physical status, no significant differences in in-hospital or ICU-mortality were observed (*p* = 0.131 and 0.112) (Table 2A, B). APACHE IV at time of ICU admission was significantly higher in patients after complicated elective surgery not surviving to ICU (*p* = 0.043) and hospital discharge (*p* < 0.001) (Table 2A, B).

**Table 2:** Perioperative risk assessment scores and (A) in-hospital mortality and (B) ICU mortality

	Elective surgery							Emergency surgery				
	Survivors			Non survivors	*p* value			Survivors		Non survivors		*p* value
*(A)*												
ASA*												
II	13			18	0.131			14		10		0.511
III	9			14				11		11		
IV	0			0				5		8		
P-POSSUM mortality**	5.22 [2.11–11.16]			3.5 [1.82–13.68]	0.951			13.59 [5.16–27.99]		41.34 [17.51–63.18]		***0.005***
APACHE IV^†^	81.00 (± 17.08)			118.73 (± 33.60)	***<0.001***			79.30 (± 27.73)		98.84 (± 21.24)		***0.007***

*(B)*												
ASA												
II	15			6	0.112			16		8		0.171
III	11			12				17		5		
IV	0			0				6		7		
P-POSSUM mortality**	4.46 [1.82–8.22]			9.80 [1.92–18.37]	0.445			16.52 [7.64–44.50]		41.02 [15.96–61.94]		***0.054***
APACHE IV^†^	90.48 (± 26.65)			114.36 (± 36.74)	***0.043***			83.06 (± 26.70)		99.33 (± 23.17)		***0.034***

Data displayed as absolute number, mean (SD) and median [IQR].

Statistically significant values are in italic and bold.

See Table 1 legend for abbreviations.

^*^N = 103 (elective 44, emergency 59), **N = 97 (elective 37, emergency 60), ^†^*N* = 84 (elective 32, emergency 52).

**Fig. 3:**
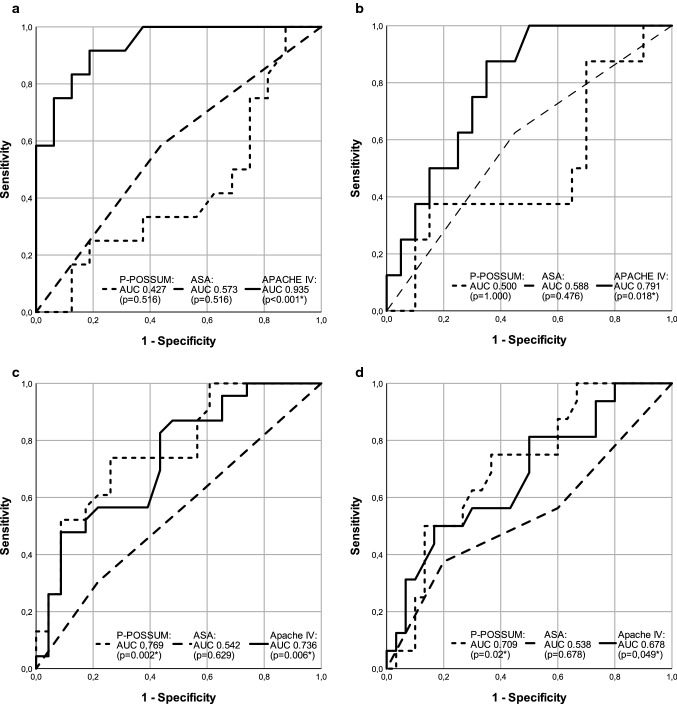
ROC curves mortality prediction by P-POSSUM, ASA and APACHE IV. **a** In-hospital mortality prediction in elective surgery. **b** ICU mortality prediction in elective surgery. **c** In-hospital mortality prediction in emergency surgery. **d** ICU mortality prediction in emergency surgery. Software: SPSS 23 (IBM Corp. Armonk, NY)

In patients with complications after elective surgery, the AUC for in-hospital mortality by P-POSSUM mortality score, ASA physical status and APACHE IV was 0.427, 0.573 and 0.935 respectively (*p* = 0.516, 0.516 and < 0.001) (Table 3A, Fig. 3a). AUC for ICU mortality after elective surgery is shown in Table 3B and Fig. 3b. All scores were well calibrated, see Table 3A, B.

**Table 3:** AUC and Hosmer–Lemeshow statistics for the prediction of (A) in-hospital mortality and (B) ICU mortality

	Elective surgery				Emergency surgery			
	AUC (95% CI)	*p* value	HL statistics	*p* value	AUC (95% CI)	*p* value	HL statistics	*p* value
*(A)*								
ASA*	0.573 (0.355–0.790)	0.516	NA^**‡**^	NA^**‡**^	0.542 (0.374–0.710)	0.629	0.015	0.904
P-POSSUM** mortality	0.427 (0.206–0.648)	0.516	8.599	0.283	0.769 (0.633–0.906)	***0.002***	16.735	***0.033***
APACHE IV^†^	0.935 (0.849–1.000)	***<0.001***	5.740	0.676	0.736 (0.592–0.710)	***0.006***	10.905	0.207
*(B)*								
ASA*	0.588 (0.352–0.823)	0.476	NA^**‡**^	NA^**‡**^	0.538 (0.352–0.723)	0.678	2.448	0.118
P-POSSUM** mortality	0.500 (0.248–0.752)	1.000	7.194	0.409	0.709 (0.557–0.862)	***0.020***	11.227	0.189
APACHE IV^†^	0.791 (0.624–0.957)	***0.018***	6.980	0.539	0.678 (0.515–0.842)	***0.049***	9.366	0.312

Statistically significant values are in italic and bold.

See Table 1 legend for other abbreviations.

*AUC* area under the curve, *95% CI* 95% confidence interval, *HL* Hosmer–Lemeshow, *NA* not applicable.

^*^N = 103 (elective 44, emergency 59), **N = 97 (elective 37, emergency 60), ^†^*N* = 84 (elective 32, emergency 52), ^**‡**^not applicable because only two available groups for analysis (i.e. ASA 2 and ASA 3).

In patients requiring ICU admission after emergency abdominal surgery ICU mortality of 34.9% and in-hospital mortality of 49.2% was observed. Median postoperative survival in patients that died during hospitalization was 14 days (Table 1). In patients after emergency abdominal surgery P-POSSUM mortality scores were significantly higher in patients who did not survive up to hospital and ICU discharge (Mann Whitney *U*, *p* = 0.005 and 0.054) (Table 2A, B) with an observed AUC of 0.769 (*p* = 0.002) for in-hospital mortality (Table 3A, Fig. 3C). ASA physical status did not discriminate between patients who did and did not survive to hospital of ICU discharge (*p* = 0.511 and 0.171) (Table 2A, B). Comparable to patients after elective surgery, APACHE IV at the time of ICU admission was significantly higher in patients not surviving to hospital discharge (*p* = 0.007) (Table 2A). The observed AUC for APACHE IV and ASA physical status in patients after emergency surgery not surviving up to hospital discharge were 0.736 and 0.542 respectively (*p* = 0.006 and 0.629) (Table 3A, Fig. 3c). Table 3B and Fig. 3d display AUC for ICU mortality after emergency surgery. All scores were well calibrated, except P-POSSUM mortality score for in-hospital mortality after emergency surgery showing a statistically significant lack of fit, see Table 3A, B.

### Comorbidity scores, frailty and mortality

Since comorbidity and frailty scores are not affected by acute illness both acute and elective patients were analysed as a single cohort. CCI, mFI and RCRI failed to identify patients who would survive to ICU or hospital discharge (Table 4).

**Table 4:** Predictability of comorbidity assessment scores of mortality

	ICU			In-hospital		
	survivors	Non survivors	*p* value	survivors	Non survivors	*p* value
Charlson comorbidity index*	3.00 [2.00–6.00]	3.00 [2.00–6.00]	0.35	3.00 [2.00–6.00]	3.00 [2.00–6.00]	0.49
Modified frailty index*	3.00 [2.00–4.00]	3.00 [1.25–4.00]	0.502	3.00 [2.00–4.00]	3.00 [1.50–4.00]	0.39
Revised cardiac risk index*	2.00 [1.00–2.00]	2.00 [1.00–2.00]	0.715	2.00 [1.00–2.25]	2.00 [1.00–2.00]	0.719

Data displayed as median [IQR].

^*^N = 107.

## Discussion

With the general population aging and a subsequent rising number of elderly undergoing surgery, elderly suffering from postoperative complications and possible critical illness requiring ICU admission increase as well [2–4]. Preoperative risk assessment is an important and necessary part of the preoperative workup and helpful in decision making [5, 6]. Reliable risk assessment remains challenging [6]. The value of perioperative risk assessment is uncertain when major complications requiring ICU admission occur. The available literature concerning the value of this perioperatively obtained outcome prediction in elderly experiencing severe complications after surgery is limited, especially in relation to ICU admission.

In this selected elderly patient population preoperative risk assessment by ASA physical status or perioperative P-POSSUM mortality scores based on parameters obtained at the outpatient clinic did not accurately predict mortality once severe septic complications occurred after elective abdominal surgery requiring an unplanned ICU admission. Both scores showed poor discriminatory ability for either in-hospital or ICU mortality. These findings emphasize the impact of severe complications on the physical reserve capacity of elderly patients and the rapid decline of vitality in this vulnerable population. The tenfold difference between predicted and observed in-hospital mortality (4.97 vs. 48.9%) and the fact that there is no relation between perioperatively determined mortality risk and actual mortality rates in these patients underlines that the assessment of physical status before surgery is no longer valid in decision making in elderly with sepsis after elective abdominal surgery.

The contrast between the predicted and observed mortality rate shows the grim prognosis of an elective abdominal operation with a complicated course in elderly patients. In the era of shared decision making it is important to discuss or reassess the willingness of a patient to undergo a burdening ICU treatment in the light of an insecure outcome. In such a discussion it is important to clarify to the patient and its relatives that the chances of a satisfactory clinical outcome may alter dramatically during the postoperative course due to the occurrence of complications.

As expected, better agreement between predicted and observed mortality rates was seen in elderly patients requiring ICU admission after emergency surgery. P-POSSUM mortality scores discriminated patients not surviving to hospital discharge with relatively good test accuracy. In these patients P-POSSUM mortality scores reflect the actual physiological status at time of surgery and not the premorbid physical function. Based on poor calibration however, P-POSSUM mortality score could not accurately predict the absolute in-hospital mortality risk. ASA physical status had no value in mortality prediction in elderly who required ICU admission after emergency surgery.

Current findings indicate that risk assessment based on the premorbid state before occurrence of acute illness is no longer reliable once complications requiring ICU admission occur. In case of ICU admission, renewed risk assessment should be performed based on the current physical state of the elderly patient. In this study, APACHE IV scores measured at the time of critical illness in both elderly after elective and emergency surgery were significantly higher in patients not surviving to hospital discharge, irrespective of the premorbid physical function of the patient. Acute disease severity scores should be calculated and used in decision making irrespective of premorbid physical capacity.

The results of this study are in line with findings in current literature. ASA physical status and P-POSSUM mortality scores of deceased and non-deceased patients after elective surgery with prolonged postoperative ICU admission were largely overlapping [20]. After emergency surgery, P-POSSUM mortality score was a reliable predictor of mortality in elderly patients [21]. Furthermore ASA physical status has been described as a poor predictor in elderly undergoing emergency surgery where APACHE scores show moderate to good discriminating value [7, 22]. Based on these previous published data and the results from this study, it can be concluded that perioperative risk assessment based on parameters obtained at the outpatient clinic is not valid in elderly with septic complications after elective surgery in need of an ICU admission with or without repeated surgery. In contrast, risk assessment based on actual physical state does seem to remain its validity as shown when risk assessment is performed at times of emergency surgery or critical illness. This emphasizes that one should reassess at every new “hit” and one should not rely on assessment performed at times of better health [23, 24].

Surgeons and critical care physicians seek better risk stratification and prediction. Current findings highlight the need of other, possibly better and more reliable, risk assessment tools. Improving the preoperative phenotyping of elderly surgical patients, for example by thorough cardiopulmonary testing and frailty assessment, might improve the insight in individual patient risk [6]. Frailty seems to be related to surgical outcome and is becoming incorporated in preoperative risk assessment and prehabilitation programs [25, 26]. Where a majority of elderly appears to suffer from undiagnosed frailty, frailty assessment might be helpful in predicting and preventing postoperative complications [27, 28]. Also regarding ICU related outcome, frailty is gaining interest where it seems to impair recovery after critical illness with increased morbidity and mortality and decreased independency and quality of life [29–35]. However, it should always be kept in mind that severe septic complications almost inescapably lead to a quick deterioration of the physical reserve of a hitherto vital and active elderly person. It may be that in this specific patient category the severity of the acute disease overwhelms the effect of a reasonable premorbid physical function. In this study no differences in frailty and chronic co-morbidities between survivors and non-survivors were identified. However, the modified frailty index was calculated retrospectively, which may have impacted its reliability. The study is further limited by a relatively small sample size that may have resulted in a type II error in comorbidity and frailty indexes although medians and ranges of comorbidity of these parameters largely overlap.

In conclusion, current perioperative risk assessment based on outpatient data is not predictive of mortality in elderly suffering from septic complications requiring ICU admission after elective abdominal surgery. However, based on current data, risk assessment with P-POSSUM and APACHE IV scores obtained at time of critical illness are predictive of mortality. Risk assessment based on premorbid functioning is not helpful and mortality risk must be reassessed based on current physical status when elderly are admitted to ICU because of postoperative septic complications. More research is needed regarding development and validation of risk assessment tools, for example incorporating frailty, that are able to predict outcome of critical illness in elderly after complicated elective surgery preferably before critical illness occurs.

## Abbreviations

APACHE: Acute Physiology and Chronic Health Evaluation
ASA: American Society of Anaesthesiologists physical status classification
AUC: Area under the curve
CCI: Charlson comorbidity index
ICU: Intensive care unit
IQR: Interquartile range
mFI: Modified frailty index
MUMC: Maastricht University Medical Centre
NICE: National Intensive Care Evaluation
PACU: Post-Anaesthesia-Care-Unit
P-POSSUM: Portsmouth Physiological and Operative Severity Score for the enumeration of Mortality
ROC: Receiver operating characteristic
RCRI: Revised cardiac risk index
SD: Standard deviation
